# Noninvasive neurological monitoring to enhance pLVAD-assisted ventricular tachycardia ablation – a Mini review

**DOI:** 10.3389/fcvm.2023.1140153

**Published:** 2023-03-08

**Authors:** Tom De Potter, Chiara Valeriano, Dimitri Buytaert, Stefaan Bouchez, Joris Ector

**Affiliations:** ^1^ Cardiovascular Center Aalst, Arrhythmia Unit, OLV Hospital, Aalst, Belgium; ^2^ Department of Advanced Biomedical Sciences, University of Naples Federico II, Naples, Italy; ^3^ Department of Anaesthesiology, OLV Hospital, Aalst, Belgium; ^4^ Department of Cardiology, University Hospitals Leuven, Leuven, Belgium

**Keywords:** ventricular tachycardia (VT), VT ablation, non invasive neurological monitoring, near-infrared spectroscopy (NIRS), electroencephalogram (EEG), percutaneous left ventricular assist device (pLVAD)

## Abstract

For critically ill patients, hemodynamic fluctuations can be life-threatening; this is particularly true for patients experiencing cardiac comorbidities. Patients may suffer from problems with heart contractility and rate, vascular tone, and intravascular volume, resulting in hemodynamic instability. Unsurprisingly, hemodynamic support provides a crucial and specific benefit during percutaneous ablation of ventricular tachycardia (VT). Mapping, understanding, and treating the arrhythmia during sustained VT without hemodynamic support is often infeasible due to patient hemodynamic collapse. Substrate mapping in sinus rhythm can be successful for VT ablation, but there are limitations to this approach. Patients with nonischemic cardiomyopathy may present for ablation without exhibiting useful endocardial and/or epicardial substrate-based ablation targets, either due to diffuse extent or a lack of identifiable substrate. This leaves activation mapping during ongoing VT as the only viable diagnostic strategy. By enhancing cardiac output, percutaneous left ventricular assist devices (pLVAD) may facilitate conditions for mapping that would otherwise be incompatible with survival. However, the optimal mean arterial pressure to maintain end-organ perfusion in presence of nonpulsatile flow remains unknown. Near infrared oxygenation monitoring during pLVAD support provides assessment of critical end-organ perfusion during VT, enabling successful mapping and ablation with the continual assurance of adequate brain oxygenation. This focused review provides practical use case scenarios for such an approach, which aims to allow mapping and ablation of ongoing VT while drastically reducing the risk of ischemic brain injury.

## Introduction

Changes in hemodynamics can be life-threatening, particularly for critically ill patients. Patients with various medical conditions may suffer from difficulties with heart contractility and rate, vascular tone, and intravascular volume; these difficulties may result in hemodynamic instability, where adequate aerobic metabolism is not maintained (1, 2). Consequently, hemodynamic support aims to aid in achieving and maintaining situationally acceptable hemodynamic function, which comprises blood pressure, cardiac output (CO), and pulmonary venous pressure. This enables maintenance of end-organ perfusion and adequate blood oxygenation, while also promoting diuresis when necessary for cases of volume overload (3).

### Forms of hemodynamic support

Traditional first line treatment for hemodynamic support includes vasopressor and inotropic drugs. While these methods are noninvasive, and typically result in increased blood pressure and CO, end-organ perfusion is often unreliable, and heart-related adverse effects can occur (3, 4). Counterpulsation by intraaortic balloon pump (IABP) may be employed to enhance the effects of inotropes, though this often adds more stress to the impaired myocardium by increasing native cardiac work and oxygen consumption (3). More recently, the development of temporary percutaneous cardiac support devices has improved the landscape of mechanical circulatory support (MCS) options. If vasopressors and inotropes are not sufficient, patients are often treated with extracorporeal bypass pumps or intracorporeal transvalvular systems. These devices are designed to reduce myocardial wall stress, stroke work, and oxygen consumption, while promoting coronary and end-organ perfusion and reducing ventricular volume and filling pressures (5). Extracorporeal bypass pumps include the TandemHeart system and venoarterial extracorporeal membrane oxygenation (VA-ECMO), while Impella remains the only current intracorporeal transvalvular system (3).

Impella is approved for use in cardiogenic shock; it has also been used in acute myocardial infarction complicated by cardiogenic shock, during high-risk percutaneous coronary intervention, cardiomyopathy with acute decompensation, postcardiotomy cardiogenic shock, off-pump coronary bypass, and more recently, ventricular tachycardia (VT) ablation (6, 7). Impella devices offer unique benefits not encountered with other methods of hemodynamic support. The transvalvular nature of Impella can enable complete unloading of the stressed left ventricle (LV) while providing blood flow at rates between 2.5 and 5.0 L/min. This drastically reduces native cardiac work and allows the damaged myocardium time to recover while increasing systemic circulation (7). The Impella acts independent of heart function and rhythm and the aortic pressure increases commensurate with the pump flow rate, causing a widening dissociation between aortic pressure and peak LV pressure (“LV-Ao uncoupling”). This unloading also results in decreased left atrial and wedge pressures (8).

Hemodynamic support has a crucial and specific benefit when employed during percutaneous ablation of VT. Mapping, understanding, and treating the particular mechanism of the arrhythmia during sustained VT without support is often not feasible, as the vast majority of patients will experience hemodynamic collapse (9). Consequently, substrate mapping in sinus rhythm was developed for VT ablation. While certainly successful to a large extent, this approach is not without limitations. Notably, in patient with diffuse substrate it may fail to delineate the extent of the diseased myocardium and the critical isthmus where ablation should be performed. Functional VT substrate mapping approaches aim to overcome this limitation; however, a standardized strategy has not yet been defined with total reproducibility and there is great variability among centers and operators performing VT ablation procedures (10). An increasing number of patients with nonischemic cardiomyopathy present for ablation, but do not exhibit useful endocardial substrate-based ablation targets. Even epicardial mapping may not reveal regions of abnormal tissue, leaving activation mapping during ongoing VT as the only viable diagnostic strategy. Additionally, a smaller proportion of patients present with near incessant VT, or VT storm, and can also ostensibly benefit from hemodynamic support during their ablation procedure (8).

### Limitations of hemodynamic support

As mentioned, each form of MCS has its own hemodynamic benefits, limitations, and related complications. IABP provides a modest increase in CO, but at the expense of increased risk for vascular stroke injury and limb ischemia. VA-ECMO offers the greatest level of CO augmentation, but also risks vascular injury, limb ischemia, bleeding, sepsis, and embolism. Impella devices offer a range of CO levels and LV support but require a large arterial cannula and carry the highest risks for vascular injury (11). Furthermore, percutaneous hemodynamic support is not a complete replacement for native cardiac function. Thus, the exact type and level of hemodynamic support required is dependent on the individual needs of the patient. Decision making should be based on the patient's need for hemodynamic support, while ensuring that the necessary levels of increased oxygenation, LV unloading, and end-organ perfusion are achieved but not exceeded, in order to avoid additional adverse effects.

There are several challenges related to percutaneous LV assist device (pLVAD) use during VT ablation, including a higher complication rate due to the need for additional vascular access, continuous anticoagulation, and interference with electroanatomical mapping systems (12). A critical risk for Impella usage is the increased likelihood of cerebrovascular accident (CVA) and ischemic stroke. Heparin and other anticoagulants are crucial for pump functionality but increase the risk of hemorrhagic events, while insufficient end-organ perfusion heightens the risk of stroke. Thus, a system should exist that comprises monitoring potential CVA and understanding the level of hemodynamic support required and the level of end-organ perfusion being achieved. Various measurements are currently employed to determine global end-organ perfusion. Low perfusion is characterized by increased serum lactate levels and an increased central-venous-arterial CO_2_ gap (*P* (v-a) CO_2_), where elevated (>6 mmHg) *P* (v-a) CO_2_ is an indicator of decreased systemic blood flow (13). However, these tools are not direct measurements and are not capable of determining effects on microcirculation, and are therefore insensitive to early oxygen depletion and organ damage.

### Noninvasive cerebral oximetry

More recently, noninvasive monitoring of cerebral tissue oxygen saturation by near-infrared spectroscopy (NIRS) has become more frequent, particularly in the context of Impella usage during VT ablation. VT is often unstable in patients in need of VT ablation, resulting in significant hemodynamic difficulties (14). Impella provides a crucially improved hemodynamic profile in these patients, improving cerebral oxygenation and thus enabling more complete VT mapping (6). However, the ideal mean arterial pressure (MAP) necessary for VT ablation patients remains unknown. Patients undergoing pLVAD-assisted VT ablation frequently experience prolonged periods of nonpulsatile flow, where the mean intravascular and cerebral perfusion pressures necessary to maintain organ and brain function may differ from conditions with pulsatile flow. Given the innate hemodynamic challenges in these patients, cerebral oximetry monitoring can detect alterations in oxygenation earlier than other commonly used, noninvasive methods or metrics (6, 15). Additionally, the electroencephalogram (EEG) is a non-invasive measurement which accurately characterizes the dynamic changes in brain function. It provides useful information regarding patient anesthetic state, as well as other facets of brain function. A sudden deterioration in the EEG pattern such as an acute onset of burst suppression may indicate cerebral hypoperfusion. Both increased brain oxygen consumption and decreased brain electrical activity reflect compromised cerebral oxygen delivery or perfusion, and early warning and intervention may prevent the incidence of neurological impairment (16).

The evidence supporting neuromonitoring for oxygen saturation is derived from numerous clinical arenas. In the cardiac surgery setting, monitoring and treatment of declining regional cerebral oxygen saturation prevented prolonged desaturations, correlated with reduced ICU visits and major organ morbidity and mortality (17), and may be associated with a lower incidence of postoperative cognitive dysfunction (18). This was mirrored in other surgical arenas, where cerebral oximetry anesthesia management in elderly patients undergoing major abdominal surgery resulted in reduced cognitive decline and hospital stays, owing to less hypoxic exposure (19). Studies have also been carried out in the electrophysiology lab, as it is known that brief episodes of circulatory arrest have fleeting but serious effects on brain function (20). Early pilot studies of NIRS cerebral oximetry usage in cases of adults with supraventricular tachycardia and VT revealed that age and left ventricular ejection fraction (LVEF) influence cerebral perfusion, underlining the benefit of NIRS monitoring in settings of induced arrhythmias (21). Cerebral spatially resolved-NIRS with high-frequency sampling was recently used to assess beat-to-beat effect of atrial fibrillation on cerebral microcirculatory perfusion; in front of a non-significant decrease in arterial blood pressure extreme events between pre- and post- electrical cardioversion, a significant difference was observed in tissue hemoglobin index values (15). This method has some limitations, as hyperthermia and norepinephrine affect skin oxygenation and can directionally influence cerebral oximetry readings (22).

**Figure 1:**
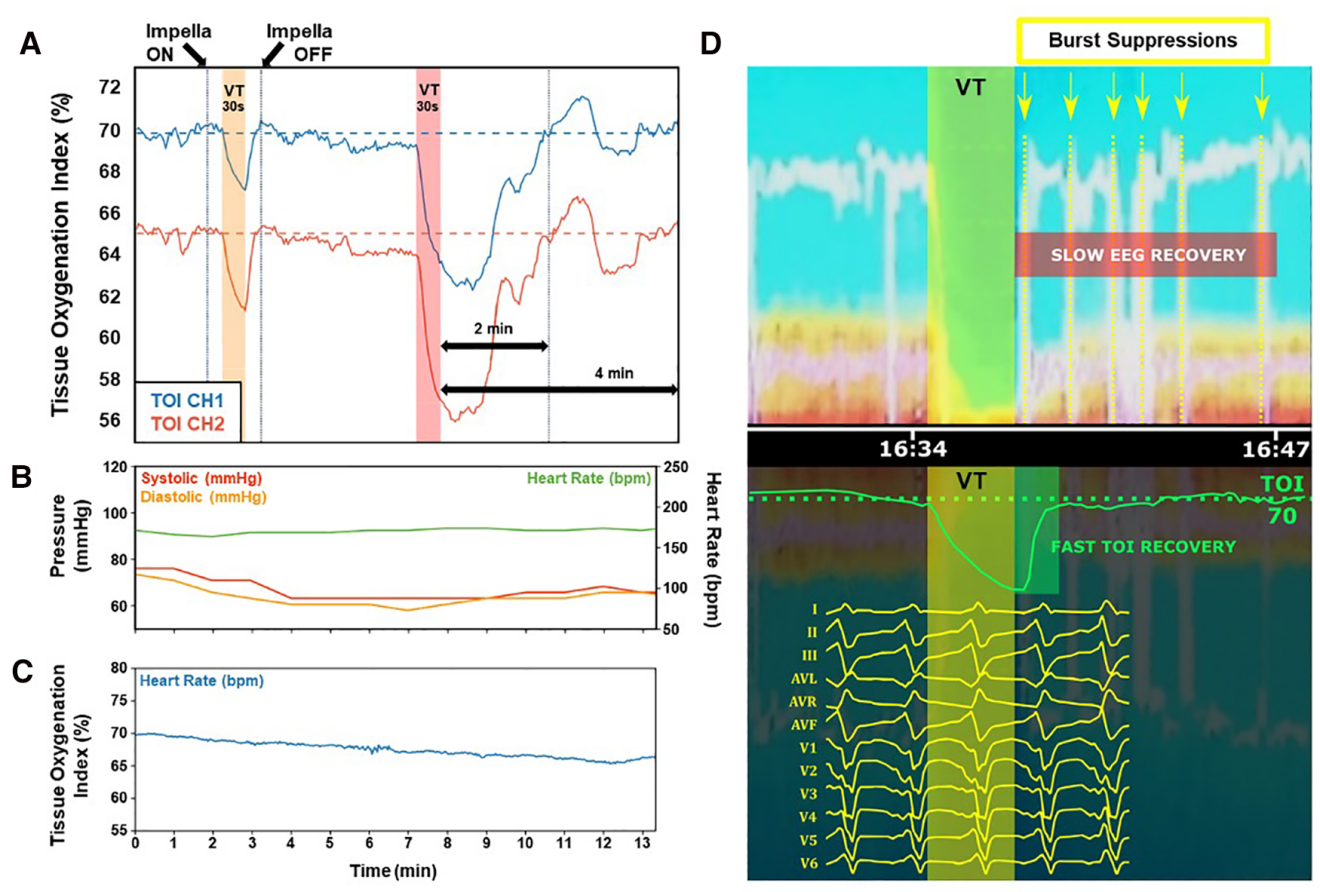
**Clinical parameters of case studies.** (**A**) Tissue oxygenation index (TOI) is significantly preserved and recovers quickly when Impella device is on during VT (yellow region). Without Impella support during VT (red region), TOI deficits are significantly more severe with subsequent instability and slower recovery. (**B**) Typical nonpulsatile flow is demonstrated during VT mapping, without clear indications of what MAP values are acceptable for end organ perfusion. (**C**) Preserved and stable TOI indicating adequate brain oxygenation demonstrates very little variation during the procedure. (**D**) A sudden deterioration of perfusion was observed despite stable VT cycle length and pressure curves after more than 30 min of mapping (bottom). EEG monitoring revealed a near complete loss of electrical brain activity, indicating a loss of adequate brain oxygenation (top). TOI recovery was near-immediate (bottom), but EEG monitoring demonstrated a burst suppression pattern (indicating suboptimal brain function) for several minutes after VT termination (top). VT, ventricular tachycardia; TOI, tissue oxygenation index.

As indicated above, pLVAD support is increasingly used in patients in need of VT ablation. Cerebral oximetric monitoring may serve as an “early warning” of decreased oxygen delivery to the brain. Figure 1 illustrates the implementation and interpretation of this monitoring in real life practice.

– In panel A, NIRS perfusion is measured on two separate channels, from which the tissue oxygenation index (TOI) is determined. Normal TOI values range from 60%–80%, and a drop of >20% from baseline values, or a value below 55%, may indicate poor oxygen delivery. Two 30 s VT episodes are simulated by rapid ventricular pacing (220 ms cycle length). During the first VT episode, Impella support is active, and only a moderate TOI drop is observed on both channels, with fast and complete recovery after cessation of VT. Conversely, during the second VT episode, Impella support is disabled and a profound drop in TOI is observed, taking 2 min to recover post-cessation of VT, and remaining unstable for an additional 2 min.
– In panel B, arterial blood pressure curves and TOI collected at the onset of VT in a clinical case are presented. Typical nonpulsatile flow with overlap of systolic and diastolic curves is observed, offering very little guidance of what MAP values are acceptable for end organ perfusion.
– Panel C shows TOI for this same VT onset, remaining within normal range with only minor drift from baseline, indicating adequate brain oxygenation during this part of the procedure.
– Panel D shows a sudden deterioration of perfusion that was observed despite stable VT cycle length and pressure curves after more than 30 min of mapping, as evinced by the sharp drop of TOI after a reasonably horizontal phase (panel D, bottom). Simultaneous EEG monitoring revealed a near complete loss of electrical brain activity, indicating a loss of adequate brain oxygenation (panel D, top); the VT was subsequently terminated by DC cardioversion (not shown). TOI recovery was near-immediate, but EEG monitoring demonstrated a burst suppression pattern (indicating suboptimal brain function) for several minutes after VT termination (panel D, top).

Percutaneous LVAD support is able to safely maintain end-organ perfusion even in the face of extended periods of hemodynamically unstable VT, enabling greater ability to execute entrainment and activation mapping. This translates into an improved hemodynamic profile, and avoidance of cerebral desaturation below a threshold of 55% (23). These observations highlight that without the benefit of NIRS cerebral oximetry and EEG monitoring, cerebral perfusion is unknown, and patient safety may be compromised. Neuromonitoring ensures that cerebral perfusion is not compromised during ongoing VT in a patient under general anesthesia, enabling clinicians to continue the procedure with confidence, or terminate the VT and/or the procedure prematurely when necessary to avoid hypoxia (6). To better illustrate this concept, we present three cases that employed neuromonitoring during VT ablation to ensure adequate cerebral perfusion and safety, which would otherwise be unknown.

### Case 1

A 51-year-old woman with a VVI implantable cardiac defibrillator (ICD) for secondary prevention after an episode of sustained monomorphic VT presented with cardiac sarcoidosis and preserved LVEF. Magnetic resonance imaging (MRI) revealed a transmural left anterior and subepicardial late gadolinium enhancement. During the patient's first procedure, a bi-ventricular voltage map revealed normal endocardial substrate. However, sustained VT was not inducible despite an aggressive stimulation protocol and pharmacological autonomic tone modulation. After 3 months, a new episode of sustained monomorphic VT was terminated by ICD shock. With an endocardial and epicardial approach confirming normal voltage maps, sustained monomorphic VT (CL of 230 ms) was induced. During VT, a drop in arterial blood pressure and loss of CO was confirmed by NIRS neuromonitoring (NIRO-200NX monitoring system). As such, the VT was terminated by electrical cardioversion and an Impella 2.5 pLVAD was implanted to enable VT activation mapping. After pLVAD placement, cerebral perfusion was immediately repristinated and patient hemodynamics stabilized. Epicardial mapping revealed a focal (concentric) activation pattern and successful ablation of the VT was performed, resulting in VT termination during radiofrequency (RF) ablation and non-inducibility (focal VT; region: lateral left-ventricle, next to the mitral annulus). There were no complications. The patient experienced an early recurrence during the first month but has since been recurrence-free for ≥2 years.

### Case 2

A 68-year-old man with a cardiac resynchronization therapy device (CRT-D) implanted for primary prevention presented with non-ischemic dilated cardiomyopathy with reduced LVEF. The patient underwent an initial endocardial ablation for VT targeting late potentials, but experienced multiple recurrent VT events. A new procedure was performed with an endocardial and epicardial approach, with no circulatory support at case onset. Mapping revealed diffuse epicardial distribution of fragmented electrograms over both ventricles that was considered too extensive for substrate ablation. After VT induction, the patient showed hemodynamic instability and loss of CO. An Impella CP pLVAD was placed to allow VT activation mapping which showed a macro-reentry with critical conduction slowing over a modest region at the epicardial right ventricular outflow tract (RVOT). Ablation at this region was performed resulting in VT termination and the RVOT area was targeted for elimination of abnormal electrograms, after which non-inducibility was achieved. VT induction testing was guided by EEG and after VT termination, TOI recovery was near-immediate, though EEG monitoring often showed a few minutes of burst suppression pattern. A year post-procedure, the patient has not experienced any recurrence.

### Case 3

A 64-year-old man with a CRT-D implanted for primary prevention presented with ischemic cardiomyopathy with reduced LVEF. The patient had two prior endocardial ablations and one endo-epicardial attempt due to recurrent VT storms with multiple ICD shocks. He was on a heart transplant waiting list due to lack of other options for arrhythmia control, despite a fairly good functional status classified as NYHA class 1 or 2. After referral for a second opinion, a new ablation was performed with an endocardial and epicardial approach with upfront Impella CP pLVAD circulatory support. Substrate mapping was performed followed by a VT induction protocol with burst pacing. The activation map revealed an endo-epicardial macro-reentry at the base of the LV, next to the mitral annulus. After ablation in this zone, other sustained VTs were still inducible, originating from the scar region of the LV (anterior-apical); these were also successfully ablated. Despite borderline MAP values, non-invasive neuromonitoring revealed sufficient end-organ perfusion throughout the procedure and eventually contributed to the procedural success (Figure 1C).

### Case report conclusions

The key messages of this case series are that the presence of a pLVAD during ablation is a determining factor for procedural success, and that noninvasive neuromonitoring enables informed decision-making. Crucial factors for decisions regarding pLVAD placement were the absence of endo-epicardial substrate (patient 1), extensive substrate and impaired LVEF (patient 2), and the clinical history of multiple VT recurrences and presentation with VT storm (patient 3). Avoiding cerebral desaturation during prolonged periods of nonpulsatile flow reduced the risk of end-organ damage and contributed to a safer and more effective ablation. The presence of EEG also provided additional information that improved the assessment of complete brain recovery after VT termination.

## Discussion

In the evolving landscape of pharmacological treatment for heart failure, more patients with ischemic, and even more so non-ischemic heart failure, are enjoying increased survival rates than ever before. These improvements have also challenged the existing VT ablation strategy of substrate mapping in sinus rhythm and have led to the identification of the limitations of this approach in the most challenging cases. These limitations present in cases where there is no identifiable useful substrate, where targeting the available substrate is not adequate for arrhythmia control, or where the substrate is too diffuse to make focal treatment by ablation a realistic option. In these scenarios, activation mapping may be considered, leveraging techniques and approaches used in supraventricular arrhythmias, provided the patient can be kept in a hemodynamic state that allows VT to exist long enough for detailed mapping. Mechanical circulatory support in the form of pLVADs can be introduced as needed during a procedure and provide enough support to make this approach realistic. Prompt identification of patients requiring prophylactic mechanical hemodynamic support may prevent periprocedural acute hemodynamic decompensation, which is associated with increased risk of mortality over follow-up (24). The PAINESD risk score has been recently developed and validated in independent cohorts as a predictor of hemodynamic decompensation and adverse outcomes in patients undergoing VT ablation and could help identify patients with advanced HF who may most benefit from preprocedural hemodynamic optimization and periprocedural mechanical hemodynamic support (25, 26). The adequacy of end organ perfusion still necessitates careful monitoring in patients carrying the largest disease burden. To that end, we have reported excellent experience with real-time monitoring of perfusion and brain function as the most critical target for perfusion.

**Figure 2:**
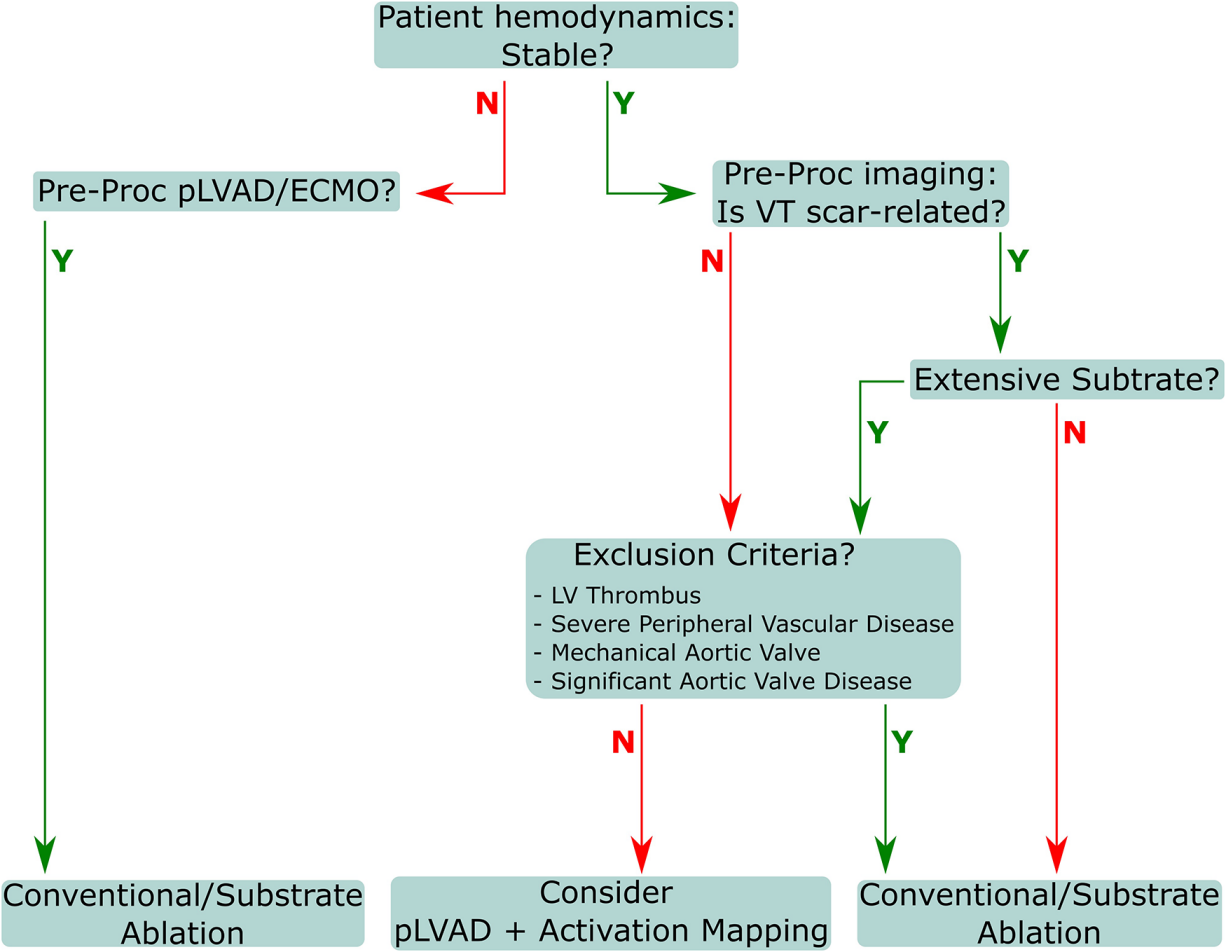
**Patient evaluation flow for VT.** VT, ventricular tachycardia; pLVAD, percutaneous left ventricular assistance device; ECMO, extracorporeal membrane oxygenation; LV, left ventricle.

All procedural indication assessments should involve input from a multidisciplinary team that includes an electrophysiologist, a heart failure specialist, and a cardiac intensivist. Subsequent decisions regarding appropriate procedural flow should involve the electrophysiologist, cardiac anesthetist, cardiac intensivist, and cardiac and vascular surgeons. The primary goal is to achieve patient weaning from the pLVAD at the end of procedure, except in cases where the patient presented as initially unstable, with pLVAD/ECMO already in place. Procedures should include closure of the access site with percutaneous devices, and as such, coordination with the surgical team is essential. Decisions regarding treatment and approach must consider patient stability and substrate conditions, as well as exclusion criteria and VT inducibility (Figure 2). Several preprocedural diagnostics are necessary to appropriately assess potential or absolute contraindications for pLVAD use. Echocardiographic visualization of the LV must be performed to exclude the presence of an LV thrombus, as a mobile thrombus may result in systemic embolization and increase the risk of device malfunction from thrombus aspiration in the inlet. Additionally, a mechanical aortic valve replacement absolutely precludes pLVAD usage for this indication.

Conversely, some contraindications are relative, and may instead require additional procedures and/or tools to facilitate safe usage in these populations. Severe aortic regurgitation is one such contraindication, as the pLVAD mechanism increases aortic pressure, which can aggravate aortic insufficiency; this may lead to significant volume loading of the LV and result in dilatation. This may be counteracted with procedures such as surgical or transcatheter aortic valve replacement (27, 28), central aortic oversewing (28), percutaneous transcatheter closure of the aortic valve (28, 29) Another relative contraindication is severe aortic valve stenosis, which can complicate placement of the catheter in the LV. Aortic valve stenosis is frequently observed in pLVAD patients, and can result in impaired device function, thromboembolism, poor LV recovery, and infection (30). Serial echocardiograms enable clinicians to continuously adjust LVAD flow speed to both allow for aortic valve opening and maximize LV decompression, ideally reducing or avoiding aortic valve stenosis or fusion (30). The presence of a ventricular septal defect (VSD) is also a relative contraindication, though VSD repair has been performed successfully with simultaneous pLVAD use (31–33), and thus pLVAD usage with careful monitoring should facilitate VT mapping in this population. Finally, as a note, while peripheral artery disease is not a contraindication, these patients are at a higher risk for numerous complications (34) and should be assessed on an individual basis for a suitable access strategy. Appropriate assessments may include angiography, intravascular ultrasound imaging (35), and computed tomography (36).

As emphasized above, in daily practice, this decision flow should include input from a multidisciplinary team. This will ensure the ideal patient approach is evaluated and selected based on patient condition, comorbidities, and potential contraindications. Subsequent to VT mapping and ablation, patients should be weaned as soon as safely possible to restore native cardiac function. Weaning assessments should also involve multidisciplinary input throughout the weaning process, to ensure all necessary conditions are continually met. Cardiac power output is a key measure of intrinsic cardiac function and must be robust (>0.6 W), and pulsatility should be preserved after reduction or cessation of inotropic or vasopressor therapy (37). Tissue perfusion must be sufficient, as indicated by an SVO2 > 60% and lactate levels <2 mmol/L. However, if the LVEF is below 20% or CO is below 0.6W, weaning should be delayed until necessary conditions are achieved (37).

## Conclusion

Combined NIRS and EEG monitoring in addition to pLVAD usage enables assessment of end-organ perfusion during VT, facilitating successful mapping and ablation. The use of pLVAD can be beneficial for all patients needing VT mapping and ablation, ameliorating the typically severe loss of CO, and neuromonitoring allows maintaining sufficient end-organ perfusion even in patients with impaired EF during long procedures. This may ultimately lead to successful VT ablation while drastically reducing the risk of ischemic brain injury.
